# Supplementary figures and images for: Characterisation of the Toxoplasma gondii tyrosine transporter and its phosphorylation by the calcium‐dependent protein kinase 3
Source: Mol Microbiol. 2018 Nov 25;111(5):1167–81. doi: 10.1111/mmi.14156 (PMC6488386; doi:10.1111/mmi.14156)

A

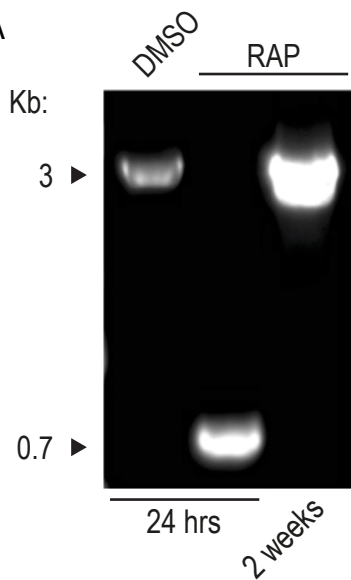

B

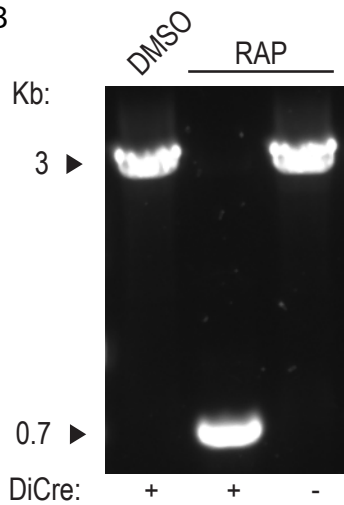

C

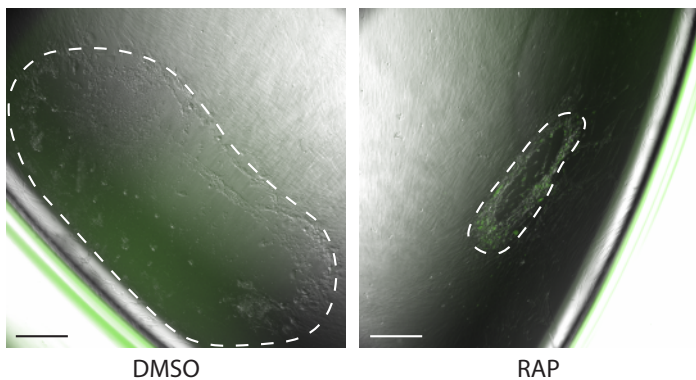

Supplement: Supplementary file 1 [file MMI-111-1167-s001.pdf]

A

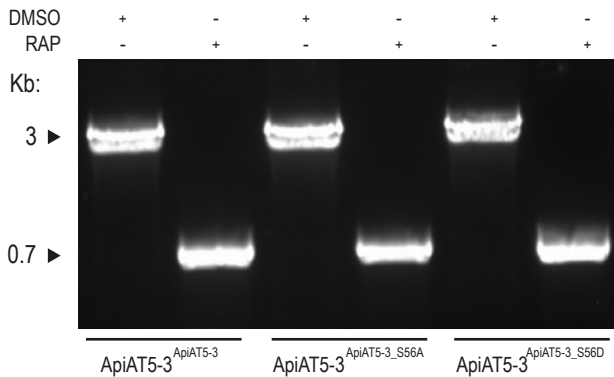

B

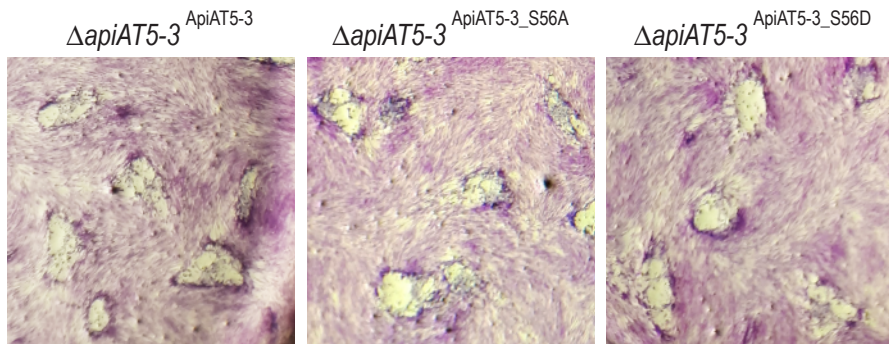

Supplement: Supplementary file 2 [file MMI-111-1167-s002.pdf]

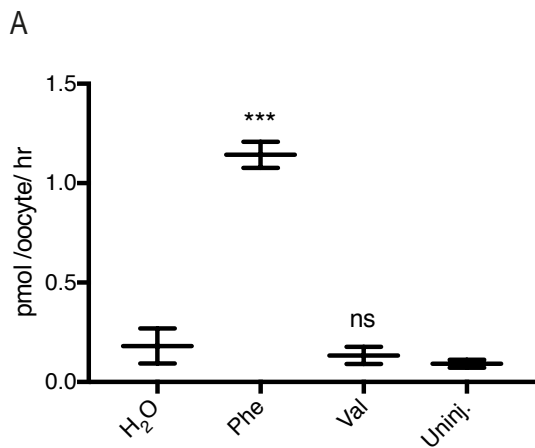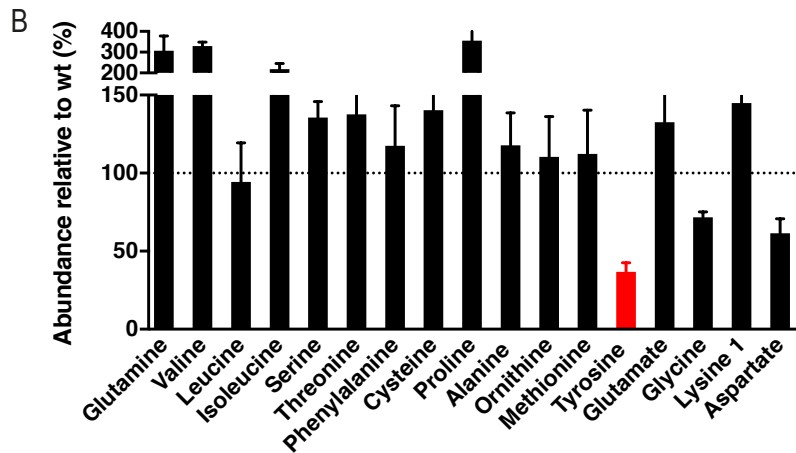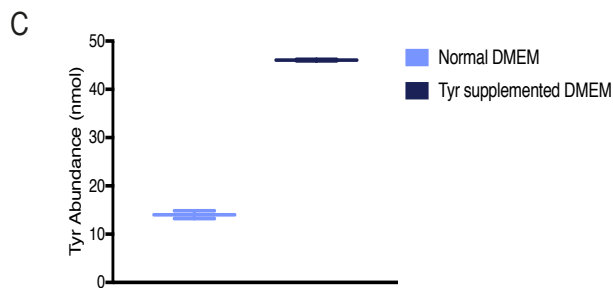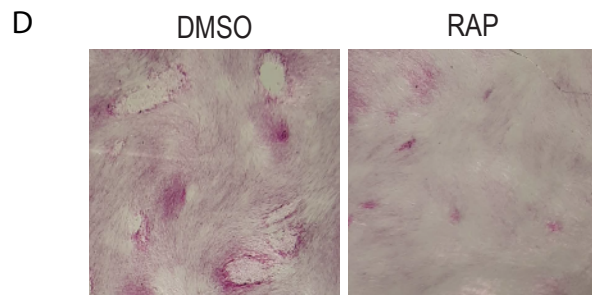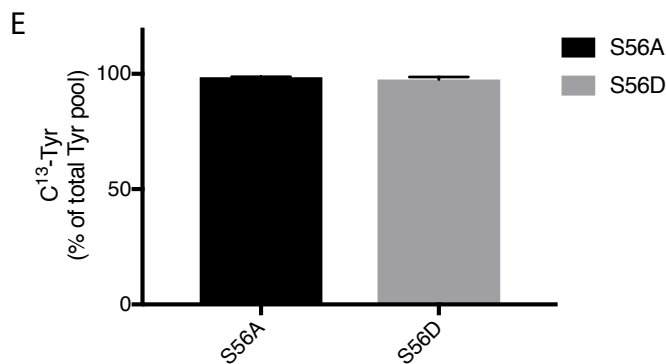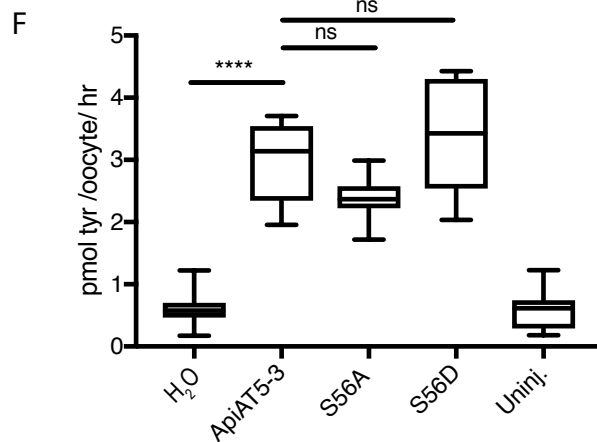

Supplement: Supplementary file 3 [file MMI-111-1167-s003.pdf]
