# Supplementary material for: Characterisation of the Toxoplasma gondii tyrosine transporter and its phosphorylation by the calcium‐dependent protein kinase 3
Source: Mol Microbiol. 2018 Nov 25;111(5):1167–81. doi: 10.1111/mmi.14156 (PMC6488386; doi:10.1111/mmi.14156)
Supplement: Supplementary file 7 [file MMI-111-1167-s007.docx]

| Strain Name | Parental Strain | Promoter | Expression Product | Description of function |
| --- | --- | --- | --- | --- |
| ApiAT5-3::HA | RH ∆*HXGPRT* | *GRA1* | *ApiAT5-3::HA* | Ectopic expression with HA tag |
| ApiAT5-3_loxP | RH ∆*ku80* diCre | *ApiAT5-3* | DMSO treated: *loxP_apiAT5-3_loxP*  RAP treated: *loxP_YFP* | Conditional knockout of ApiAT5-3 |
| ApiAT5-3_loxP^dDiCre^ | RH ∆*ku80* diCre | *ApiAT5-3* | DMSO/ RAP treated: *loxP_apiAT5-3_loxP* | Non-excisable ‘dead DiCre’ *ApiAT5-3* control |
| ApiAT5-3_loxP^ApiAT5-3^ | RH *∆ku80* ApiAT5-3_loxP | *ApiAT5-3/ UPRT* | Endogenous locus: *loxP_apiAT5-3_loxP*  UPRT locus: *apiAT5-3* | Merodiploid strain:  Floxed ApiAT5-3 in wt locus that is removed upon RAP treatment; complementing wt- ApiATS-3 in UPRT locus |
| ApiAT5-3_loxP^ApiAT5-3_S56A^ | RH *∆ku80* ApiAT5-3_loxP | *ApiAT5-3/ UPRT* | Endogenous locus: *loxP_apiAT5-3_loxP*  UPRT locus: *apiAT5-3_S56A* | Merodiploid strain:  Floxed ApiAT5-3 in wt locus that is removed upon RAP treatment; complementing phospho-dead ApiATS-3 in UPRT locus |
| ApiAT5-3_loxP^APIAT5-3_S56D^ | RH *∆ku80* ApiAT5-3_loxP | *ApiAT5-3/ UPRT* | Endogenous locus: *loxP_apiAT5-3_loxP*  UPRT locus: *apiAT5-3_S56D* | Merodiploid strain:  Floxed ApiAT5-3 in wt locus that is removed upon RAP treatment; complementing phospho-mimetic ApiATS-3 in UPRT locus |
| ∆*apiAT5-3*_loxP^ApiAT5-3^ | RH *∆ku80* ApiAT5-3_loxP | *ApiAT5-3/ UPRT* | Endogenous locus: *loxP_YFP* and  UPRT locus: *apiAT5-3* | Clone of RAP-treated ApiAT5-3_loxP^ApiAT5-3^  which has the floxed copy removed |
| ∆*apiAT5-3*_loxP^ApiAT5-3_S56A^ | RH *∆ku80* ApiAT5-3_loxP | *ApiAT5-3/ UPRT* | Endogenous locus: *loxP_YFP* and  UPRT locus: *apiAT5-3_S56A* | Clone of RAP-treated ApiAT5-3_loxP^ApiAT5-3_S56A^  which has the floxed copy removed |
| ∆*apiAT5-3*_loxP^APIAT5-3_S56D^ | RH *∆ku80* ApiAT5-3_loxP | *ApiAT5-3/ UPRT* | Endogenous locus: *loxP_YFP* and  UPRT locus: *apiAT5-3_S56D* | Clone of RAP-treated ApiAT5-3_loxP^ApiAT5-3_S56D^  which has the floxed copy removed |
